# Efficient depolymerization of polyethylene terephthalate (PET) and polyethylene furanoate by engineered PET hydrolase Cut190

**DOI:** 10.1186/s13568-022-01474-y

**Published:** 2022-10-26

**Authors:** Fusako Kawai, Yoshitomo Furushima, Norihiro Mochizuki, Naoki Muraki, Mitsuaki Yamashita, Akira Iida, Rie Mamoto, Takehiko Tosha, Ryo Iizuka, Sakihito Kitajima

**Affiliations:** 1 Graduate School of Environmental and Life Sciences, Okayama University, 1-1-1 Tsushima-Naka, Kita-Ku, Okayama, 700-8530 Japan; 2 Toray Research Center, Inc, 3-7 Sonoyama 3-Chome, Otsu, Shiga 520-8567 Japan; 3 Faculty of Agriculture, Kindai University, 3327-204, Nakamachi, Nara, Nara 631-8505 Japan; 4 Division of Clinical Nutrition, Faculty of Nutrition, Kobe Gakuin University, 518 Arise, Ikawadani-Cho, Nishi-Ku, Kobe, Hyogo 651-2180 Japan; 5 RIKEN SPring-8 Center, 1-1-1, Kouto, Sayo, Hyogo 679-5148 Japan; 6 Graduate School of Science, The University of Tokyo, 7-3-1 Hongo, Bunkyo-Ku, Tokyo, 113-0033 Japan; 7 Graduate School of Science and Technology, Kyoto Institute of Technology, 1 Hashigami-Cho, Matsugasaki, Sakyo-Ku, Kyoto, Kyoto 606-8585 Japan

**Keywords:** Cut190 variant, PET hydrolase, Micronization, Milling, PET package, PET bottle

## Abstract

The enzymatic recycling of polyethylene terephthalate (PET) can be a promising approach to tackle the problem of plastic waste. The thermostability and activity of PET-hydrolyzing enzymes are still insufficient for practical application. Pretreatment of PET waste is needed for bio-recycling. Here, we analyzed the degradation of PET films, packages, and bottles using the newly engineered cutinase Cut190. Using gel permeation chromatography and high-performance liquid chromatography, the degradation of PET films by the Cut190 variant was shown to proceed via a repeating two-step hydrolysis process; initial endo-type scission of a surface polymer chain, followed by exo-type hydrolysis to produce mono/bis(2-hydroxyethyl) terephthalate and terephthalate from the ends of fragmented polymer molecules. Amorphous PET powders were degraded more than twofold higher than amorphous PET film with the same weight. Moreover, homogenization of post-consumer PET products, such as packages and bottles, increased their degradability, indicating the importance of surface area for the enzymatic hydrolysis of PET. In addition, it was required to maintain an alkaline pH to enable continuous enzymatic hydrolysis, by increasing the buffer concentration (HEPES, pH 9.0) depending on the level of the acidic products formed. The cationic surfactant dodecyltrimethylammonium chloride promoted PET degradation via adsorption on the PET surface and binding to the anionic surface of the Cut190 variant. The Cut190 variant also hydrolyzed polyethylene furanoate. Using the best performing Cut190 variant (L136F/Q138A/S226P/R228S/D250C-E296C/Q123H/N202H/K305del/L306del/N307del) and amorphous PET powders, more than 90 mM degradation products were obtained in 3 days and approximately 80 mM in 1 day.

**Graphical Abstract:**

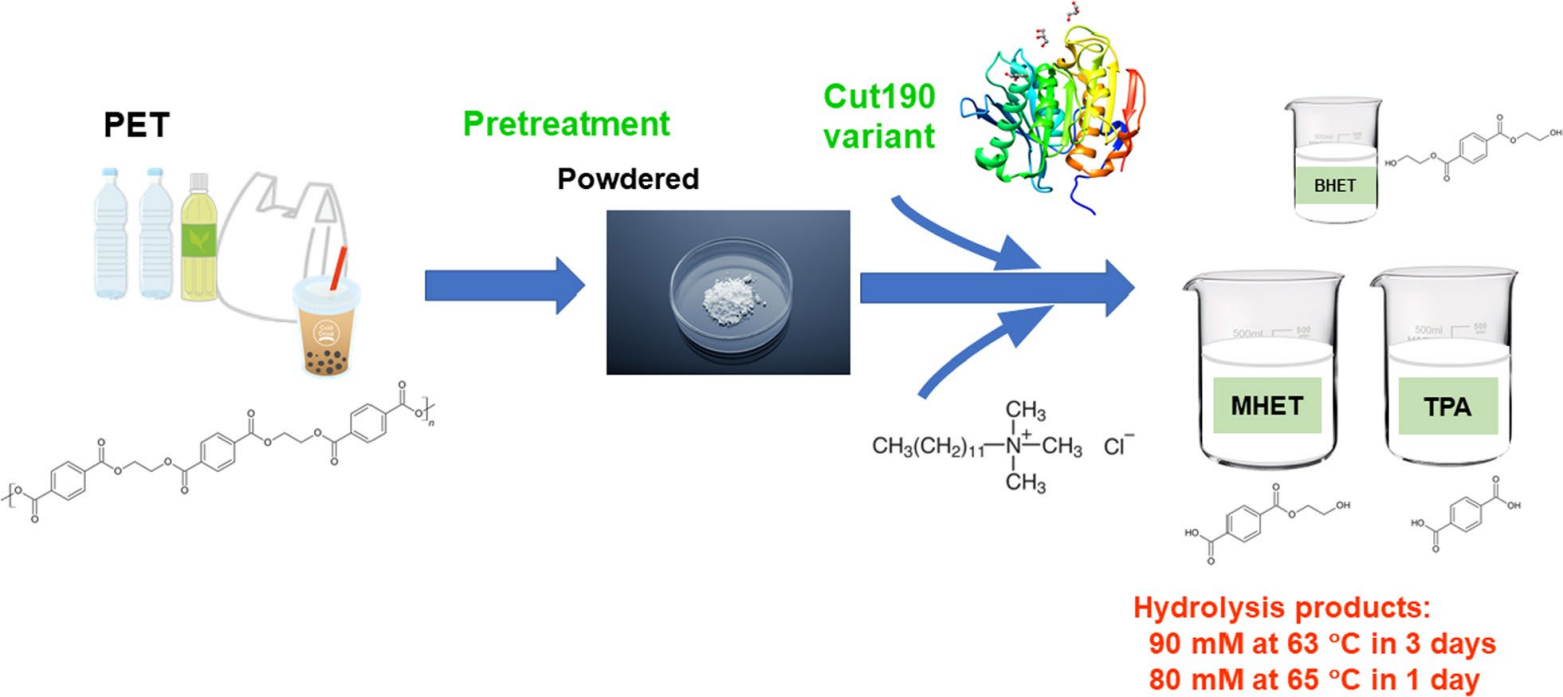

**Supplementary Information:**

The online version contains supplementary material available at 10.1186/s13568-022-01474-y.

## Key points

1. The increased surface area of PET promotes its hydrolysis by Cut190, supporting the surface erosion mechanism by a two-step process (endo-type scission of a polymer chain and exo-type hydrolysis of depolymerized fragments).
2. Dodecyltrimethylammonium chloride functioned as a binding module between Cut190 and PET surface, showing the higher hydrolysis rate at 65 °C than at 70 °C in the absence of the detergent.
3. The best performing Cut190 variant produced more than 90 mM degradation products at 63 °C in 3 days and approximately 80 mM at 65 °C in one day, using amorphous PET powders. Additionally, polyethylene furanoate was highly hydrolyzed at 63 °C in 3 days by the Cut190 variant.

**Supplementary Information:**

The online version contains supplementary material available at 10.1186/s13568-022-01474-y.

## Introduction

Of the 6300 million tons of plastic waste generated between 1950 and 2015, only 9% was recycled (Geyer et al. 2017). Polyethylene terephthalate (PET) is one of the major plastics used worldwide and its global production is amounted to 82 million metric tons per year (Singh et al. 2021). Historically, PET had been considered recalcitrant to biodegradation, due to the presence of the aromatic dicarboxylic acid terephthalate in PET, which confers a high glass transition temperature (*T*_g_) (75–80 °C in air but lowered to 65–70 °C in water), thereby requiring a PET hydrolase with high thermostability and activity at 70 °C for degradation (Kawai et al. 2019; Thomsen et al. 2022). Since the first report on PET hydrolase from a thermophilic actinomycete, *Thermobifida fusca* (Müller et al. 2005), many PET hydrolases and their degradation abilities have been documented (Kawai 2021). However, high-level candidate degraders with the ability to produce degradation products such as mono/bis(2-hydroxyethyl) terephthalate (MHET/BHET) and terephthalate (TPA) on an industrial scale are limited and need further approaches to design efficient PET hydrolases as well as to achieve ultra-high-throughput screening from novel resources (Wei et al. 2022). Among already known candidates, cutinases from thermophilic actinomycetes (*T. fusca*, *T. cellulosilytica* and *Saccharomonospora viridis*) (Wei et al. 2019; Zhang et al. 2022; Emori et al. 2020), a leaf-branch compost metagenome-derived cutinase (LCC) (most probably derived from *Chloroflexi* species; Xi et al. 2021) (Tournier et al. 2020; Zeng et al. 2022) and a thermophilic fungus cutinase from *Humicola insolens* (commercialized) (Castro et al. 2019) have been reported to possess a thermostability at more than 70 °C and to accumulate high millimolar level of degradation products. Tournier et al. (2020) reported an efficient process for PET recycling, based on two strategies using amorphized and particulate PET with engineered variants of the LCC. Most recently, a novel PET hydrolase was cloned from a compost metagenome (Sonnendecker et al. 2022) and its variant indicated the comparable activity to the LCC variant (Pfaff et al. 2022). In addition, PET hydrolases are cloned even from human saliva metagenome (Eiamthong et al. 2022). Thermostable mutants of PETase from mesophilic *Ideonella sakaiensis* (Yoshida et al. 2016) have been documented so far. HotPETase attained the highest *T*_m_ value of 82.5 °C and showed comparative activity at 60–70 °C to the LCC variant (Bell et al. 2022). On the other hand, FAST-PETase with *T*_m_ value of 67.1 °C reported the better performance at 50 °C, compared to LCC and its variant (Lu et al. 2022). We isolated a thermophilic actinomycete, *S. viridis* AHK190, from compost and cloned a cutinase-like enzyme, Cut190, possessing the unique feature that the thermostability and activity are controlled by Ca^2+^, in a concentration-dependent manner (Kawai et al. 2014). The increase in Cut190 thermostability by Ca^2+^ is attributed to the increased enthalpy change, which is partially compensated by the increased entropy change (Inaba et al. 2019). The three major Ca^2+^-binding sites of Cut190/S226P/R228S (Cut190*) were determined by X-ray crystallography (Numoto et al. 2018), and the role of each Ca^2+^-binding site was determined by site-directed mutagenesis (Oda et al. 2018). The introduction of a new disulfide bond into one of the Ca^2+^-binding sites contributed to an increase in the melting temperature (*T*_m_) value and PET-hydrolyzing activity of the Cut 190* variant (Oda et al. 2018). In addition, the deletion of the three C-terminal amino acids of Cut190* further increased its thermal stability (Senga et al. 2020). These findings indicate that Cut190 variants can be used to efficiently hydrolyze PET.

As described previously (Kawai et al. 2019, 2020), PET hydrolysis is highly dependent on the physicochemical properties of PET, as well as the activity and thermostability of PET hydrolases. It is therefore important to understand the mechanism of enzymatic PET degradation and develop efficient hydrolysis processes with highly thermostable and active hydrolases and PET pre-processing. In this study, from the analysis of the mass distribution of PET and degradation products, we verified the catalytic mechanism of PET hydrolase as surface erosion. Based on the presumed degradation pattern of PET (surface erosion), we attempted to process PET plastics (films, packages, and bottles) by micronization to expand the surface area, thereby making PET materials readily degradable by PET hydrolases. Additionally, a cationic surfactant was employed to increase the enzyme activity at a lowered temperature (65 °C) than at 70 °C (the optimal temperature in the absence of the surfactant). Meanwhile, we introduced additional mutations on the Cut190 variants reported earlier (Senga et al. 2020; Emori et al. 2020), indicating the novel insights into the mutational points. It is noteworthy that the Cut190 variant can hydrolyze polyethylene furanoate, a possible PET alternative.

## Materials and methods

### Materials

A 0.25-mm thick film of amorphous PET (PET-GF) was obtained from Goodfellows Cambridge, Ltd. (Tokyo, Japan). The PET package (approximately 0.6-mm thick; PET-S) for PC peripherals (Sanwa Supply Inc., Okayama, Japan) (PET-S) was also used for degradation tests, like the material used in previous studies (Oda et al. 2018; Sulaiman et al. 2014). Amorphous PET films (NOACRYSTAL V and NOACRYSTAL R) and PET bottle flakes were kindly supplied by RP TOPLA Ltd. (Osaka, Japan). All the films, post-consumer bottles, and packages were cut into pieces (approximately 5 mm × 5 mm) and further homogenized in water using a homogenizer (Physcotron NS-20CG/20P, Microtec Co., Ltd., Chiba, Japan) (PET suspension). The conditions for homogenization were below 14,500 rpm for 90 s, which was repeated 50 times, using appropriate amounts of PET samples (0.3–0.5 g) per 15 ml of Milli-Q water in a 50 ml Falcon tube (for particle sizes, see Additional file 1: Fig. S1). Amorphous PET pellets (raw materials used for processing into various PET products) were milled into powder particles, (i) using the Cryogenic Sample Crusher JFC-300 (Japan Analytical Industry Co., Ltd., Tokyo, Japan) after pre-freezing in liquid nitrogen (powder 1), or (ii) first with Cutting Mill SM2000 and then with Ultra Centrifugal Mill ZM200 (Vender Scientific Co., Ltd., Tokyo, Japan) after pre-freezing in liquid nitrogen (powder 2), or (iii) with Wonder Blender WB-1 (Osaka Chemical Co., Ltd., Japan) without pre-freezing (powder 3) (for particle sizes, see Additional file 1: Fig. S2). The physical characteristics of the films, packages, and powder forms were characterized using differential scanning calorimetry (DSC) (Additional file 1: Table S1). BHET was purchased from Tokyo Chemical Industry Co., Ltd. (Tokyo, Japan). MHET was synthesized as previously described (Oda et al. 2018). Polyethylene furanoate (PEF) was synthesized, according to the method described by Pellis et al. (2016) (Additional file 1: Scheme S1). The molecular weights of PET and PEF were measured using gel permeation chromatography (GPC), on a Prominence LC system (Shimadzu Corporation, Kyoto, Japan) equipped with an RI detector using hexafluoroisopropanol as the mobile phase at 40 °C with a flow rate of 0.2 ml/min. The sample was dissolved in the mobile phase, filtered through a 0.5 µm-pore size membrane, and 20 μl of each sample was applied to two columns of TSK gel GMHHR-H(S) (7.8 mm ID × 30 cm; Tosoh Corporation, Tokyo, Japan). The molecular weights were calibrated using the polymethylmethacrylate standards. Other chemicals were obtained from commercial sources.

### Site-directed mutagenesis and expression and purification of mutant enzymes

Cut190*/Cut190** mutants used in this study are listed in Table 1. The variants were generated using site-directed mutagenesis as described previously (Oda et al. 2018; Senga et al. 2020; Emori et al. 2020), and the gene sequences encoding Cut190**SS/L136F/Q138G and Cut190**SS/L136F/Q138G/F255I were synthesized by GenScript Japan Ltd. (Tokyo). The PreScission protease hydrolysis site was introduced just after the N-terminal polyhistidine tag in all the variants. All the genes were ligated into pQE80L and expressed in *Escherichia coli* Rosetta-gami B(DE3), and the enzyme variants were purified, as described previously (Senga et al. 2020; Emori et al. 2020). To remove the polyhistidine-tag, PreScission protease (Turbo 3C, Accelagen, San Diego, CA, USA or HRV3C, Takara, Shiga, Japan) was added to the purified enzyme with a polyhistidine-tag and incubated at 4 °C for 16 h, after which the protein was purified using a Ni-nitrilotriacetic acid column (Qiagen, Hilden, Germany) and a HiTrap Q FF column (Cytiva, Marlborough, MA, USA), as described previously (Senga et al. 2020; Emori et al. 2020). The *T*_m_ value of each variant was determined by measuring the intensity change in the circular dichroism signal at 222 nm, as described previously (Oda et al. 2018). SDS-PAGE analysis revealed that the purities of the enzymes were greater than 90%. The protein concentration was spectrophotometrically determined at 280 nm using a molar absorption coefficient of 4.02 × 10^4^ M^−1^ cm^−1^.

**Table 1:** Cut190 mutants used in this study

Mutant	Mutation	References
Cut190*	S226P/R228S	Oda et al. (2018)
Cut190**	S226P/R228S/K305del/L306del/N307del	Senga et al. (2020)
Cut190*SS	Q138A/S226P/R228S/D250C-E296C/ Q123H/N202H	Emori et al. (2020)
Cut190**SS	Q138A/S226P/R228S/D250C-E296C/ Q123H/N202H/K305del/L306del/N307del	This study
Cut190**SS/L136F	L136F/Q138A/S226P/R228S/D250C-E296C/ Q123H/N202H/K305del/L306del/N307del	This study
Cut190**SS/L136F/Q138G	L136F/Q138G/S226P/R228S/D250C-E296C/ Q123H/N202H/K305del/L396del/N307del	This study
Cut190**SS/L136F/Q138G/F255I	L136F/Q138G/S226P/R228S/D250C-E296C/ Q123H/N202H/F255I/K305del/L396del/N307del	This study

### Degradation tests of PET and PEF samples

The reaction mixture for PET hydrolysis contained 100 mM HEPES–NaOH buffer (pH 8.5 and 9.0), 2.5 mM CaCl_2_, 24% glycerol_,_ and approximately 2 μM Cut190* derivative. PET films were cut into pieces of 6 mm ∅ and incubated in a 1-ml reaction mixture. Appropriate amounts of PET suspension or PET powder were also used, in addition to films. Incubation was performed at different temperatures (63–70 °C) with shaking at 110 rpm. Aliquots were withdrawn from the reaction mixture supernatant at different intervals, diluted with the same volume of methanol, and their absorbance at 240 nm was measured. Degradation products such as BHET, MHET, and TPA were confirmed using high-performance liquid chromatography (HPLC), as described previously (Hantani et al. 2018), with monitoring at 240 nm. The amounts of MHET and TPA produced in the reaction mixture were calculated from their respective standard curves. BHET was detected in trace amounts. The photometric absorption at 240 nm showed a good correlation with the HPLC measurements at 240 nm, although the values from absorbance were 5–10% higher than those obtained from HPLC (Additional file 1: Table S2). Therefore, absorption at 240 nm was used for general measurements of the reaction products.

The PEF samples were used in powder form. The reaction mixture (500 μl) contained 100 mM HEPES–NaOH buffer (pH 9.0), 2.5 mM CaCl_2_, 24% glycerol, approximately 2 μM Cut190*SS, and approximately 10 mg of PEF. After incubation at 63 °C for 3 days with shaking, the reaction was stopped by adding the same volume of methanol and filtered as described above, and the filtrate was analyzed using HPLC with detection at 265 nm. 2,5-Furandicarboxylic acid (FDA; Waken Holdings Co., Ltd., Kyoto, Japan) was used as the standard.

The measurements were performed in duplicate or triplicate.

## Results

### Mechanism of PET degradation

**Fig. 1:**
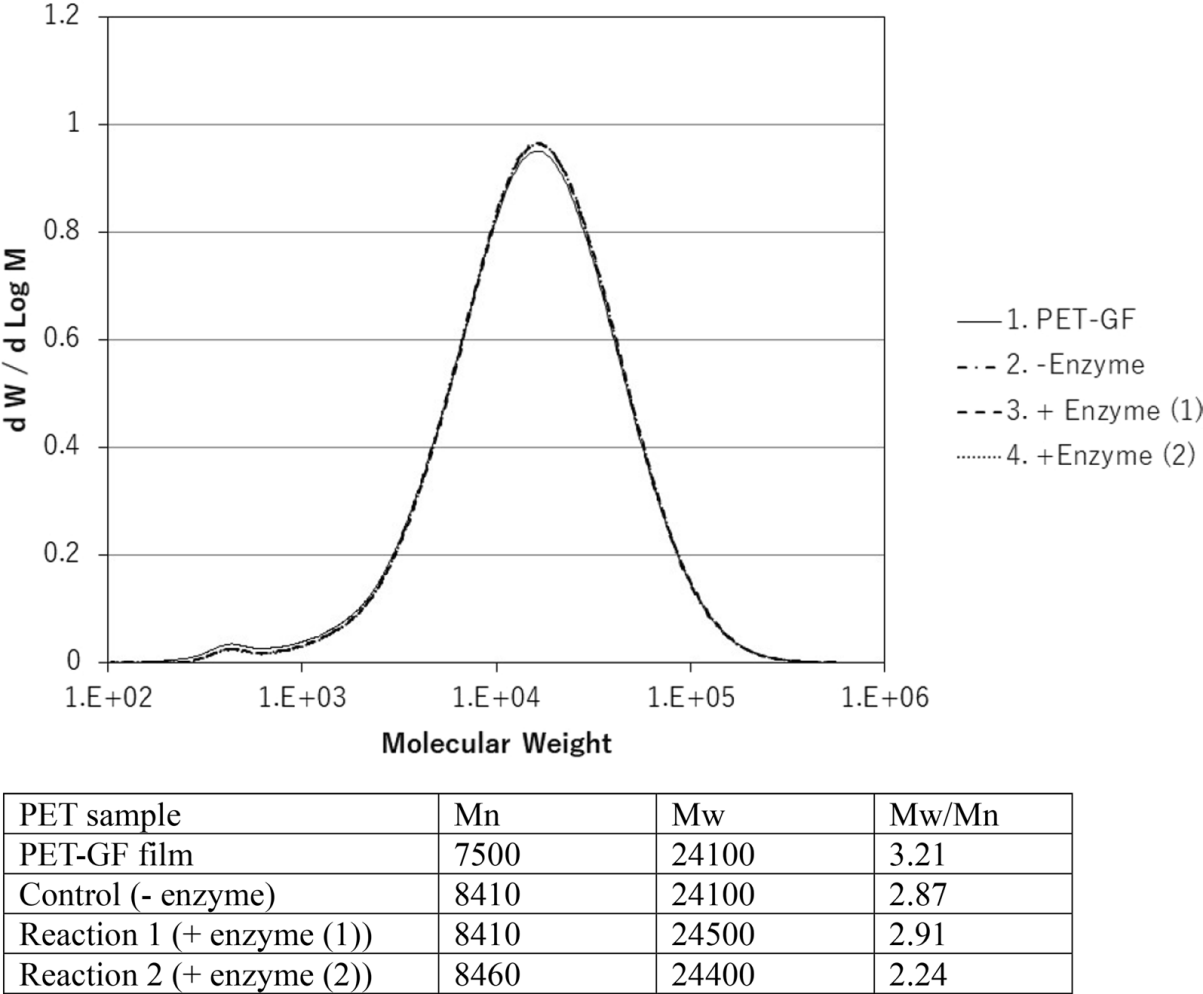
Molecular weight before and after enzyamatic hydrolysis. Reactions (1 ml) were performed at 70 °C for 3 days using Cut190*SS, one piece of 6 mm ∅ PET-GF film and 100 mM HEPES buffer (pH 8.5), as described in “Materials and methods”. Degradation rates of reactions 1 and 2 were 33.6 and 31.4%, respectively. Mn: number-average molecular weight. Mw: weight-average molecular weight

The hydrolysis of PET is considered to proceed at random via endo-type degradation (Eberl et al. 2009), which is expected to generate decreased molecular weights if degradation occurs for all molecules, as observed in polyvinyl alcohol degradation (Kawai & Hu 2009). However, no substantial change in molecular size was found with PET hydrolyzed by Cut190*SS (Fig. 1), although the degradation rate was calculated to be over 30%. This suggests that only the PET molecules on the surface layer were fragmented (surface erosion) (Müller et al. 2005; Müller 2006; Ronqvist et al. 2009). Depolymerization only at the surface does not affect the molecular size of the bulky polymer, probably because the depolymerized small molecules readily move away from the surface, leaving the main PET body intact. HPLC analysis confirmed TPA and MHET as the major reaction products from PET, and only a trace amount of BHET was detected during the reaction time, as previously reported (Herrero Acero et al. 2011; Barth et al. 2016; Oda et al. 2018). Our previous results indicated that BHET is readily hydrolyzed to MHET and TPA by Cut190* (Hantani et al. 2018). As the release of HPLC-detectable products (BHET, MHET and TPA) was proportional to the reaction time and the total amount of products were well in accordance with the decrease in weight (Ronqvist et al. 2009; Kawai et al. 2014), it is reasonable to assume that these products are mainly formed by the exo-type hydrolysis. Together, these findings suggest that PET depolymerization proceeds via surface erosion, in which the first endo-type hydrolysis occurs at random on the surface molecules, and then depolymerized products such as BHET, MHET, and TPA are produced by exo-type hydrolysis from the fragmented surface molecules. As the hydrolysis rate of MHET is far slower than that of BHET (Hantani et al. 2018), MHET persists longer than BHET; however, it is finally converted to TPA.

### Factors affecting the degradability of PET

The extent of PET hydrolysis was affected by the nature and pH of the buffers used (Additional file 1: Fig. S3). As Cut190 requires Ca^2+^ ions for its activation and stabilization, phosphate buffer cannot be used. Among HEPES, bicine, and glycine–NaOH buffers at pH 9.0, HEPES showed the highest activity, which was also higher than that of HEPES at pH 8.5. Therefore, HEPES buffer (pH 9.0) was used for further hydrolysis experiments. High product concentrations (mainly MHET and TPA) led to a reduction in pH because of insufficient buffer concentration, resulting in a decrease in PET hydrolysis, as the optimal pH of Cut190 is on the alkaline side (Kawai et al. 2014). A product level lower than 50 mM requires at least 100 mM HEPES buffer, with higher product concentrations requiring higher buffer concentrations (150 mM for 70–80 mM product and 200 mM for 80–100 mM product) to maintain an alkaline pH for longer incubation. The positive correlation between the buffer concentration and the enzymatic performance of a PET hydrolase has also been reported (Pfaff et al. 2022).

**Fig. 2:**
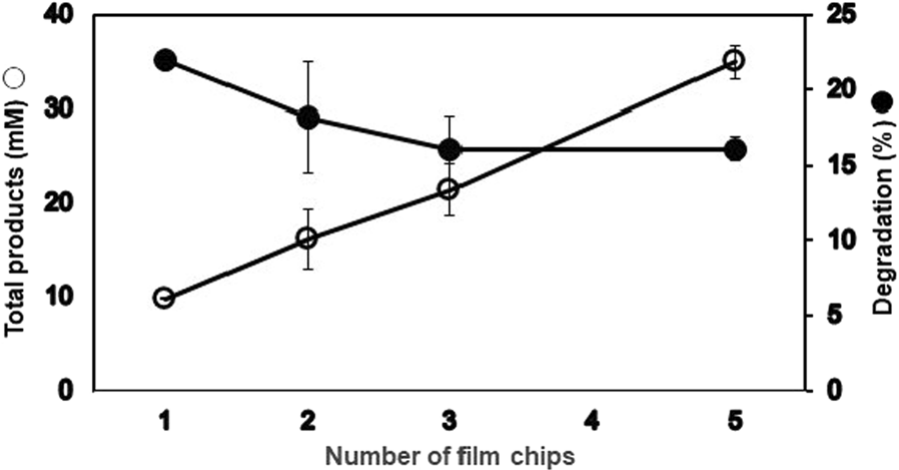
Effect of numbers of 6 mm ∅ PET films. Reactions were performed at 70 °C for 3 days, using Cut190*SS, 100 mM HEPES (pH 8.5) and 6 mm ∅ PET-GF film (approximately 9.5 mg/each corresponding to 49.4 μmoles as monomer unit/each) in a 1-ml reaction mixture, as described in “Materials and methods”

As suggested above, PET hydrolysis occurs in layers, and it is expected that widening the surface area can accelerate the degradation. When the number of 6 mm ∅ film pieces was increased (Fig. 2), the product concentrations increased, indicating that higher surface area promoted the enzyme reaction. To further increase the surface area, we prepared powder samples using different methods (powders 1–3), as described in “Materials and methods”. The degradation of the powdered form of amorphous PET was two-fold more than the film at approximately the same weight, except for powder 3, supporting the finding that the larger the surface dimension, the higher the degradation (Table 2). Compared with powder 1 (prepared with pre-freezing), powder 3 (prepared without pre-freezing) showed approximately 50% degradation, suggesting that friction heat generated during milling of PET pellets without pre-freezing must have caused physical aging on the surface of powder 3, as suggested by Wei et al. (2019). Therefore, pre-freezing is required to prevent undesirable aging of PET powder by the generated heat.

**Table 2:** Comparison of degradabilities of film and powder forms

PET	Crystallinity (%)	Weight (mg)	Total products (mM)	Degradation (%)
NOACRYSTAL R (0.6 mm-thick)	0	22.4 ± 0.2	38.3 ± 2.6	32.9 ± 1.6
Powder 1	2	20.1 ± 0.1	74.6 ± 0.92	71.7 ± 0.85
Powder 2	5	20.2 ± 0.1	74.2 ± 3.1	70.4 ± 2.5
Powder 3	0	20.1 ± 0.3	35.9 ± 4.9	34.6 ± 4.7

The reactions were performed at 63 °C for 3 days, as described in “Materials and methods”, using Cut190*SS and film (two chips/ml) and powder forms (approximately 20 mg/ml)

**Fig. 3:**
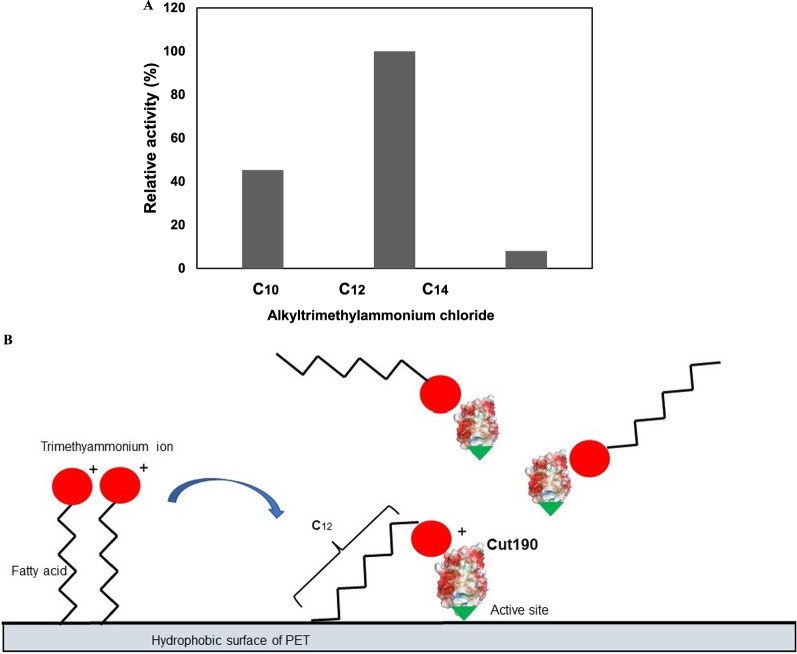
Effect of surfactants on PET hydrolysis. **A** The reactions were performed at 65 °C for 2 days, using Cut190*SS, 100 mM HEPES buffer (pH 8.5), 30 ppm (90 μM) surfactant and one piece of 6 mm ∅ PET-GF film, as described in “Materials and methods”. Surfactants used were decyltrimethylammonium chloride (C_10_), dodecyltrimethylammonium chloride (C_12_), and tetradecyltrimethylammonium chloride (C_14_). The values were shown as relative values, that for C_12_ being 100%. **B** Image for binding of dodecyltrimethylammonium chloride on Cut190**SS and PET film

We then hypothesized that enhanced adsorption of the enzyme on the PET surface can promote the enzyme reaction. Furukawa et al. (2018, 2019) reported that a negatively charged surfactant promoted PET hydrolysis by *Is*PETase, and a positively charged surfactant was effective for Tf_Cut2, by increasing the binding of these enzymes on the PET surface. We evaluated the effect of differently charged surfactants with the same fatty acid chain length (C_12_) on enzyme adsorption. For comparison, we employed the same concentration (30 ppm; approximately 90 μM) of surfactants as used by Furukawa et al. (2019). As shown in Additional file 1: Fig. S4, the addition of the cationic surfactant dodecyltrimethylammonium chloride promoted the reaction by approximately 30%, although the nonionic surfactant slightly inhibited the enzyme reaction, and the anionic surfactant slightly enhanced the reaction. We then tested other trimethylammonium chloride surfactants with shorter and longer fatty acids (C_10_ and C_14_), but dodecyltrimethylammonium chloride was the best additive to increase the adsorption of Cut190 to the PET surface (Fig. 3A). The surfactant probably meditates the appropriate distance between the PET surface and the active cleft of the Cut190 structure to improve enzyme binding to PET, as illustrated in Fig. 3B.

### Micronization improves enzymatic recovery of monomer products from post-consumer PET materials

As the expanded surface area promoted PET degradation, we attempted to micronize amorphous PET pellets or films and post-consumer PET products such as packages and bottles. In contrast to amorphous PET pellets, PET films, including flat films, shaped packages, and bottles (already stretched during the manufacturing process) cannot be sufficiently powdered. These PET films, however, were homogenized into small pieces using a homogenizer, as described in “Materials and methods”. Homogenization of PET products are proposed here for the first time for application to PET products with different shapes and crystallinities (different hardness), providing a novel means for evaluating the degradability of PET films. Although homogenization did not significantly change the crystallinity and molecular weights of PET samples, it changed the amorphous states; the rigid amorphous region (RAF) was increased, and the mobile amorphous fraction (MAF) consequently decreased (Additional file 1: Table S3). The change in the RAF/MAF ratio was small in bottle pellets but rather large in amorphous films. The friction of metal blade with PET during homogenization can generate heat and switch MAF to RAF, the effect of which is more severe in the soft amorphous films than in the hard bottle flakes. While amorphous PET plain films (PET-GF is often used) are known to be degraded by various PET hydrolases (Ronqvist et al. 2009; Kawai et al. 2014; Barth et al. 2016; Oda et al. 2018; Sonnendecker et al. 2022), they are not used as-is for any practical purpose except in research experiments. The amorphous PET package exhibited the degradation level like that of the amorphous PET film as the homogenized suspension prepared from them. The suspension prepared from PET bottle flakes showed lower degradability than that prepared from the amorphous package, but considerably higher than that of the original flakes (trace level) (Table 3). These results indicate that crystallinity, MAF/RAF ratio, and surface area are the major limiting factors for enzymatic PET hydrolysis.

**Table 3:** Comparison of degradabilities of PET suspensions from different PET sources

PET suspension	Concentration^a^		Total products^a^	Degradation
	(mg/ml)	(mM)	(mM)	(%)
PET-S	6.9	35.8	9.02 ± 0.76	25.2 ± 2.1
Waste PET bottle flakes	9.0	46.8	6.51 ± 0.09	13.9 ± 0.2
Cap for a drinking cup^b^	7.3	37.8	9.78 ± 1.48	25.9 ± 3.9

The reactions were performed with Cut190*SS at 60 °C (PET-S) or 65 °C (others) for 6 days, as described in “Materials and methods”

^a^As monomer units. One milligram of PET corresponds to 5.2 μmoles of monomer unit

^b^Food package 1 in Additional file 1: Table S1

**Fig. 4:**
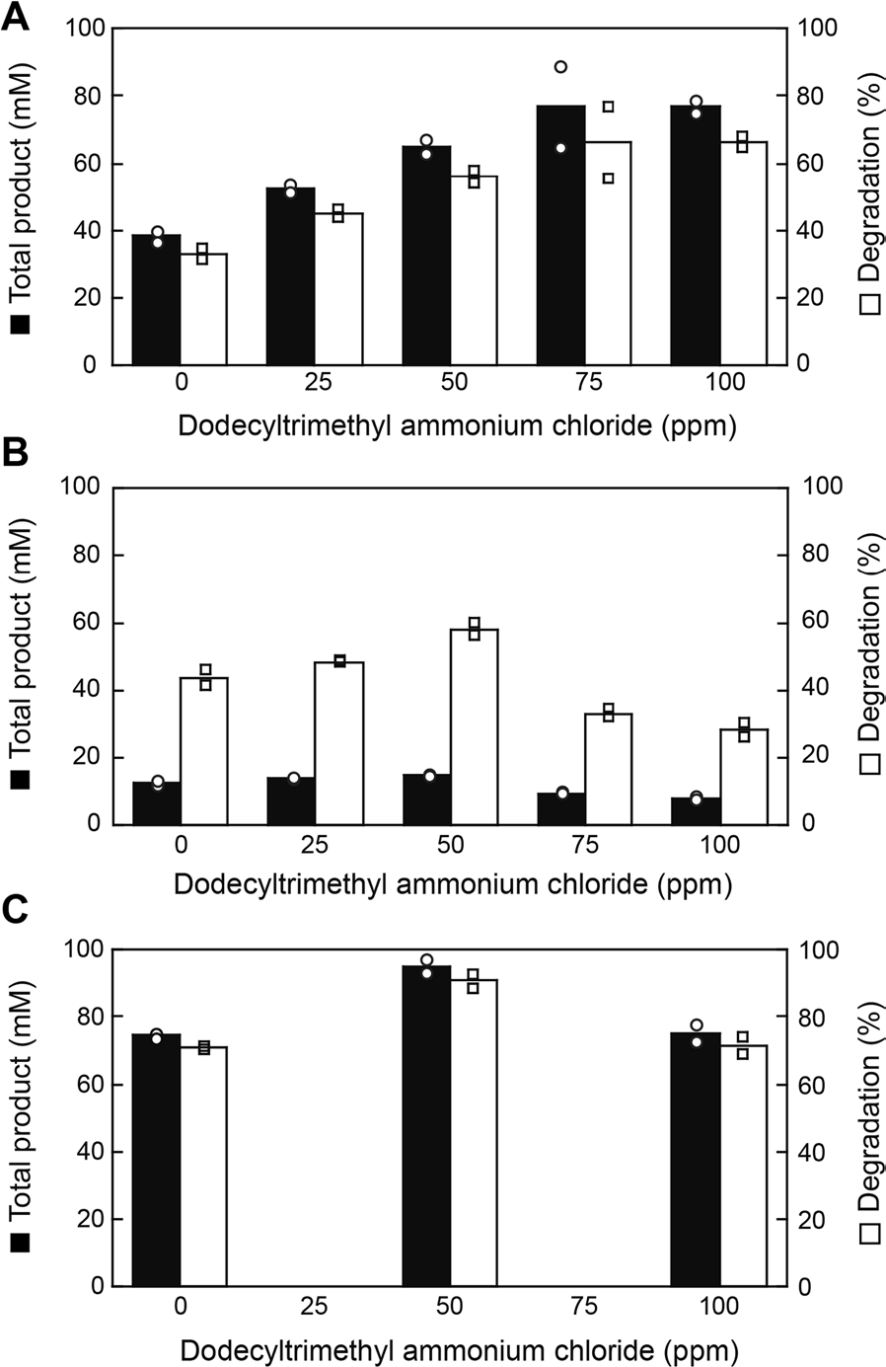
Effect of surfactant concentrations on amorphous PET film and amorphous PET powder. **A** Two pieces (6 mm ∅; 116 μmoles/ml as monomer unit) of amorphous PET film (Nonacryl V): **B** Homogenized PET bottle flakes (28.5 μmoles/ml as monomer unit): **C** Powder 2 (105 μmoles/ml as monomer unit). The reactions were performed at 63 °C for 3 days in a 1-ml reaction mixture, using Cut190*SS under the standard reaction conditions except that buffer concentration in **A** (50–100 ppm) (160–310 μM) and **C** (0–100 ppm) (0–310 μM) was raised to 150 mM. The reactions were performed in duplicate. Variations in values are indicated by open circles for total products and open squares for degradation (%)

The optimum cationic surfactant concentration to maximize PET degradability was determined for PET film, PET suspension, and PET powder (Fig. 4). Although powder 2 (C) has more than twice the surface area of the film (A) (Table 2), it required a lower optimal concentration of the surfactant. In addition, although the concentration of PET in the homogenized PET bottle pellet (B) is approximately one-fourth that of (A) and (C), the optimal concentration of the surfactant is approximately the same as those for (A) and (C). Therefore, the different PET forms might exhibit varying surface affinities of (A)–(C) for the surfactant with the shape/size or the surface microstructure being related to the binding of the surfactant on the polymer surface and the enzyme. Notably, the homogenized PET bottle pellet (B) showed approximately 60% degradation with 50 ppm dodecyltrimethylammonium chloride, the value of which is more than the amount of MAF (29%), suggesting significant degradation of RAF (43%) (see Additional file 1: Table S3). Powder 2 (C), with particle sizes of a few μm to 10 μm in length, generated over 90 mM of total products with over 80% of degradation. Taken together, surface dimensions, surface properties, and amorphous states of PET, as well as the appropriate surfactants to facilitate enzyme binding on the PET surface, can contribute to PET hydrolysis.

### Engineering of Cut190

Among the Cut190 variants reported to date, Cut190*SS has been shown to be the best performing enzyme, retaining high activity at 70 °C (PET glass transition temperature) (Oda et al. 2018). In another study (Senga et al. 2020), a variant with the three C-terminal amino acids deleted (Cut190**) showed better thermostability than Cut190*. Based on the structural analysis (Miyakawa et al. 2014), we first focused on Q138 and found that the Q138A mutation in Cut190*SS enhanced its activity towards PET (Oda et al. 2018). Tournier et al. (2020) reported that mutating Y127 in LCC (corresponding to Q138 of Cut190) to glycine (smaller than alanine) improved the thermostability of the enzyme. Chen et al. (2022) compared Q92 mutants of *T. fusca* cutinase (corresponding to Q138 of Cut190), resulting in the higher activity of Q92G than Q92A. Cui et al. (2021) reported the efficiency of L136F/Q138Y (expressed as corresponding positions in Cut190) introduced in PETase from *Ideonella sakaiensis* (*Is*PETase) (DuraPETase). Most recently, Pfaff et al. (2022) constructed L136F/Q138Y mutant of PES-H1 as the best performing variant. Although the importance of the Q138 position in PET hydrolases has no doubt, type I (TfCut2, Cut190 and LCC) and type II (*Is*PETase, PES-H1) hydrolases (Eiamthong et al. 2022) showed different replacement effects. Considering these results, we made several Cut190 variants, as listed in Table 1 and compared their PET hydrolyzing activities (Table 4). In the presence of dodecyltrimethylammonium chloride, increasing the reaction temperature of Cut190*SS till 65 °C promoted the reaction, but the reaction rate decreased at 67 °C, although Cut190*SS exhibited high activity at 70 °C (Oda et al. 2018). Cut190**SS showed higher activity than Cut190*SS, indicating that deletion of the three C-terminal amino acids is effective, not only for Cut190* but also for Cut1190*SS. Alanine appeared to be better than glycine at position 138 (in Cut190**SS/L136F and Cut190**SS/L136F/A138G, respectively), although the difference in activity was not significant. As the expression level of Cut190**SS/L136F/Q138G/F255I was tremendously decreased, additional mutation (F255I) is disadvantageous for the Cut190.

**Table 4:** Comparison of PET hydrolyzing activities

Mutant	Total products (mM)	Degradation (%)	*T*_m_ Ca^2+^	
			0 mM	2.5 mM
Cut190*	61.5	59.1	56.3	67.7
Cut190**	71.7	68.9	60.7	71.7
Cut190*SS	69.9 (75.1)	67.2 (72.3)	82.1	83.3
Cut190**SS	76.6	73.7	n.d	86.6
Cut190**SS/L136F	93.8	90.2	84.2	86.2
Cut**SS/L136F/Q138G	89.1	85.8	83.7	84.7*
Cut**SS/L136F/Q138G/F255I	− (94.9)	− (91.3)	82.7	83.5

The reaction mixture contained HEPES–NaOH buffer (pH 9.0), 2.5 mM CaCl_2_, 24% glycerol, 50 ppm dodecyltrimethylamine chloride and approximately 2 μM Cut190* or its derivative. The concentrations of the buffer were 100 mM for Cut190* and Cut190**, and 150 mM for others. Preincubation was performed at room temperature for 10–20 min with PET powder 2 (20 mg/ml) in the reaction mixture except enzyme and CaCl_2_. Then each enzyme and CaCl_2_ were added, and the incubation was started at 63 and 65 °C with shaking for 3 days. Values at 65 °C are shown in parentheses. All the experiments were performed in duplicate

^*^With 25 mM Ca^2+^. No difference was observed in the absence and the presence of 2.5 mM Ca^2+^. Nano-differential fluorometry (Nano Tempa Tycho NT.6, M&S TechnoSystems, Inc., Osaka, Japan) showed the same behavior against Ca^2+^, although *T*_m_ values were 91.5 and 91.4 °C with 0 and 2.5 mM Ca^2+^, respectively

**Fig. 5:**
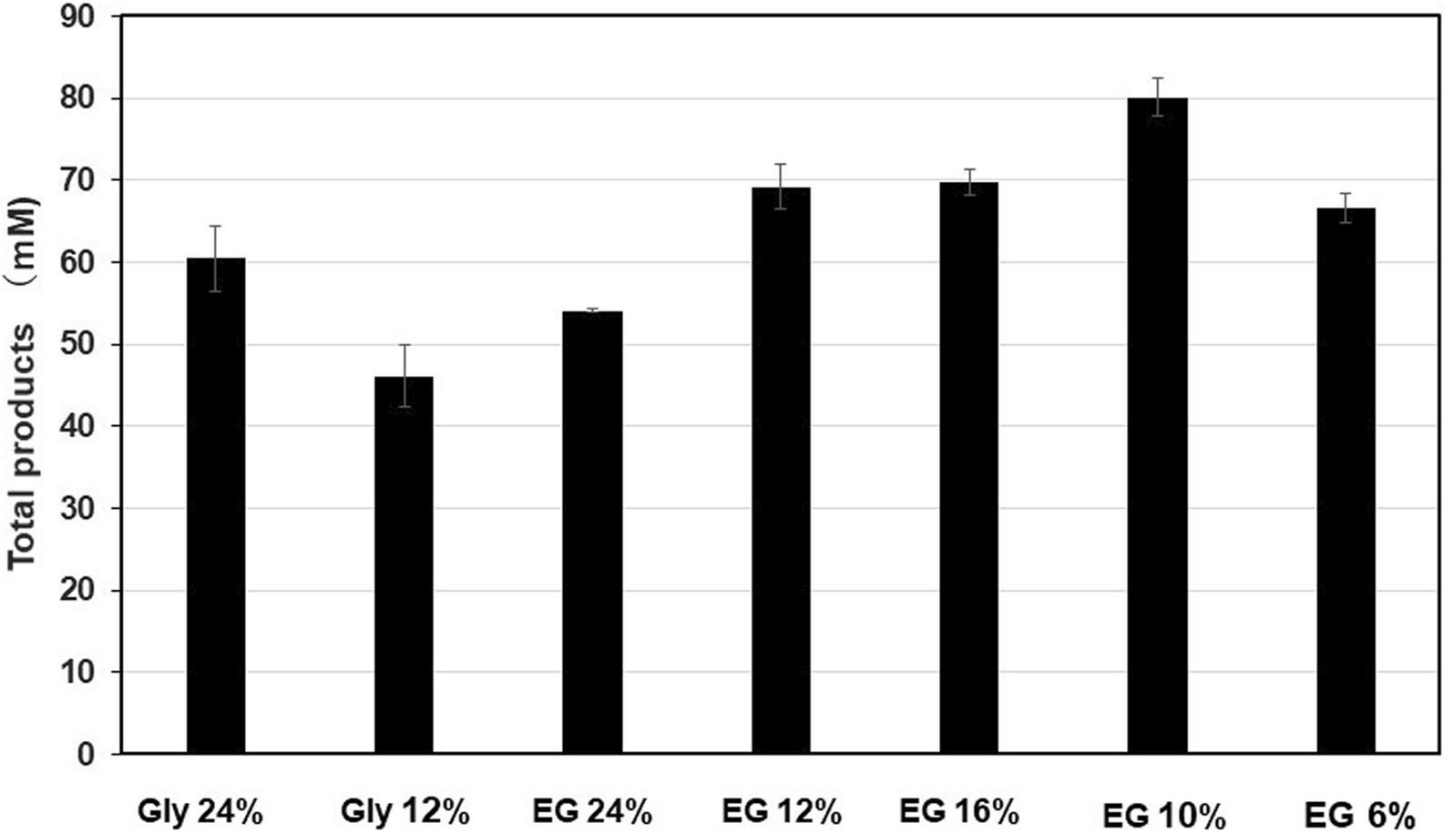
Effect of glycerol and ethylene glycol on the enzyme activity. The reactions were conducted with PET powder 2 (20 mg/ml) using Cut190**SS at 65 °C for 1 day under the assay conditions described in “Materials and methods” expect that glycerol or ethylene glycol were used at the concentrations indicated and dodecetyltrimethylammonium chloride was added at 50 ppm

We used 24% glycerol to keep the enzyme activity for longer time, as described in “Materials and methods”. Reduction of glycerol concentration or replacement of glycerol with ethylene glycol were evaluated, as shown in Fig. 5. Compared with the activity at 24% glycerol, the activity at 12% glycerol was decreased. Although the activity at 24% ethylene glycol was approximately two-thirds of that at 24% glycerol, decreased concentrations (6–16%) of ethylene glycol enhanced the activity by more than 10%. As ethylene glycol is one of the hydrolytic products from PET, replacement of glycerol with ethylene glycol should be better for the application of the Cut190 to bio-recycling of PET.

### Hydrolysis of PEF

The components of PEF are ethylene glycol and 2,5-furan dicarboxylic acid, of which the latter can be produced from carbohydrates (Munoz de Diego et al. 2011). The production of 2,5-furan dicarboxylic acid is far less expensive than that of terephthalate. Therefore, PEF is expected to be a substitute for PET (Pellis et al. 2016), although it might be difficult to replace all PET products with PEF. Cut190 has a broad substrate specificity towards various polyesters (aliphatic, aliphatic*-co*-aromatic, and PET) (Kawai et al. 2014). We therefore synthesized PEF (Additional file 1: Scheme S1) and tested its degradability using Cut190*SS. The results indicated that Cut190-type enzymes can recognize PEF as a substrate (Table 5), suggesting the potential application of Cut190 variants for future bio-recycling of various polyester materials. In contrast to the PET results, dodecyltrimethylammonium chloride inhibited the hydrolysis of PEF. PEF1 with higher weight-average molecular weight (Mw) and number-average molecular weight (Mn) was hydrolyzed more than PEF 2 with lower Mw and Mn, which agrees with the report of Pellis et al. (2016).

**Table 5:** Hydrolysis of PEF by Cut190 variant

	Mw; Mn (Mw/Mn)	Control^a^ Total products (mM)	Enzyme reaction		
			FDA (mM)	Second peak^b^ (mM)	Total products (mM)
PEF1	74,000;27,900 (2.65)	2.55	27.9	27.7	55.6
PEF2	21,300;9170 (2.32)	10.4	30.2	14.1	44.4

Cut190*SS was incubated at 63 °C for 3 days under the same conditions of PET except that 20 mg/ml of PEF (100 mM as monomer unit) was used. Control reactions were performed without the enzyme. Measurements of products were conducted by HPLC, as described in “Materials and methods”. Calculation was done, based on the peak area of FDA with different concentrations. Each measurement was performed in duplicate and expressed as average values

^a^FDA was detected, but the second peak^b^ was negligible

^b^Probably hydroxymethylfuranoate (Pellis et al. 2016)

All data generated or analyzed during this study are included in this published article and its additional information files.

## Discussion

**Fig. 6:**
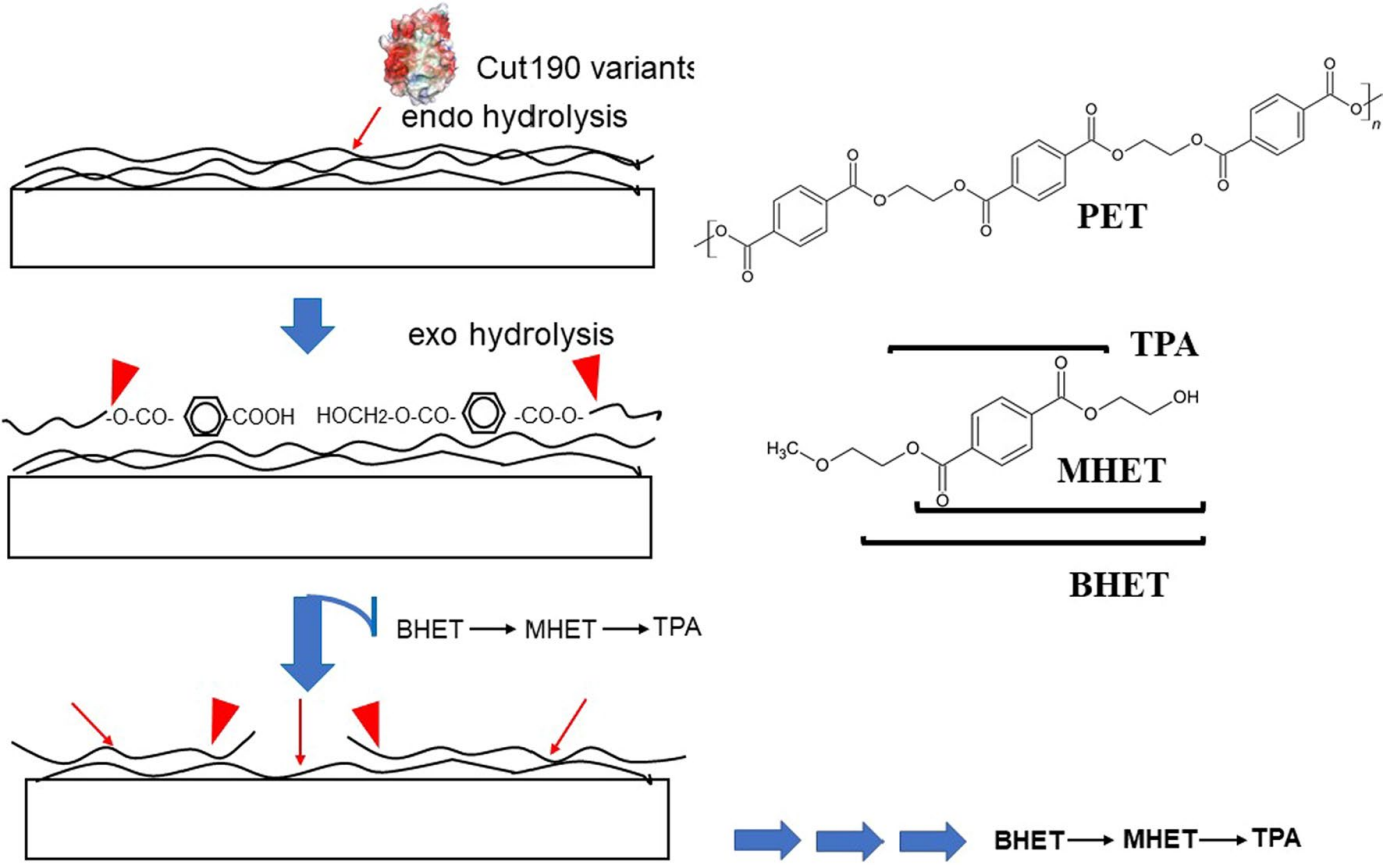
Concerted depolymerization process by endo-type and exo-type hydrolyses

Although the amorphous PET film was degraded by over 30%, almost no shift in molecular weights was observed for the remaining film, as shown in Fig. 1. The attack of the enzyme on a PET molecule proceeds via endo-type hydrolysis (Eberl et al. 2009). Herrero Acero et al. (2011) and Thumarat et al. (2015) suggested an endo-type scission mechanism using a PET model compound, bis(benzoyloxyethyl)terephthalate. In addition, BHET, MHET, and TPA have been identified as degradation products of PET (Herrero Acero et al. 2011; Barth et al. 2016; Wei et al. 2016; Oda et al. 2018), which suggests exo-type hydrolysis from the ends of the polymer chain. Taken together, endo-type and exo-type hydrolyses must proceed concurrently during PET hydrolysis. Each polymer molecule is fragmented on the surface of the polymer block and removed one by one, leaving the block of non-depolymerized molecules. Even if fragmented molecules remain on the surface of the PET block, they are readily released from the surface, or their content is negligible and does not affect the entire molecular size. This is quite different from the degradation pattern of polyvinyl alcohol only by endo-type degradation, where the polymer is water-soluble, and the molecular size of the polymer decreases rapidly because the polymer molecules are solved, dispersed, and uniformly susceptible to enzyme attack (Kawai and Hu 2009). As PET is solid, only the surface molecules are susceptible to enzyme attack, but most of the molecules are not exposed to enzyme attack unless the upper surface is removed after fragmentation. Once an ester bond is hydrolyzed on the surface by the enzyme, MHET/BHET could be produced by exo-type hydrolysis from the ends of fragmented molecules (more attackable by the enzyme), as illustrated (Fig. 6). Sufficiently depolymerized molecules are released from a PET block or removed by washing before GPC analysis, resulting in no change in the entire molecular size of the remaining block (Fig. 1). Therefore, the hydrolysis pattern of PET is a mixed type of endo-type and exo-type hydrolyses by a single PET hydrolase. Thomsen et al. (2022) suggested that the initial random, endo-scission action of enzymes immediately does not release detectable soluble products, but once a sufficient number of cleavage points has been achieved, the continued enzymatic action results in release of detectable hydrolysis products, using LCC variant and DuraPETase. Wei et al. (2019) also proposed the same degradation mechanism, using TfCut2. Therefore, the concerted reaction mechanism by endo-scission and exo-scission is considered to be common to PET hydrolases. Notably, thermostable PET hydrolases, especially from actinomycetes, can hydrolyze BHET and MHET in addition to PET, in contrast to *Is*PETase, which does not hydrolyze MHET to TPA and requires an MHET-hydrolyzing enzyme to be added for complete PET degradation (Yoshida et al. 2016; Palm et al. 2019).

The increase in the surface area of PET resulted in its increased degradation, either as film or particle. Therefore, micronization is assumed to be an effective strategy for PET bio-recycling (Gamerith et al. 2017; Tournier et al. 2020). However, careless micronization caused undesirable aging by heat generated during the process, increasing RAF as found with powder 3 (Additional file 1: Table S1). Wei et al. (2019) reported that RAF is recalcitrant for biocatalytic degradation, in contrast to that MAF is readily degraded. Gamerith et al. (2017) reported that semi-crystalline PET powder (24% crystallinity) from bottle pellets was hydrolyzed higher than amorphous PET powder (12% crystallinity) from a Goodfellows film, probably due to that both materials were crushed without pre-freezing and amorphous film must have decreased MAF, compared to bottle pellets, as shown in this study (Additional file 1: Table S3). As elucidated by Thomsen et al. (2022), crystallinity and glass transition temperature surely influence the enzymatic degradation of PET. On the other hand, Brizendine et al. (2022) concluded that amorphization of PET is necessary pretreatment step for the reaction by the LCC variant, but particle size reduction may not be required. As cryomilling of amorphous PET film apparently increased crystallinity, thereby decreasing MAF (data not shown by Brizendine et al (2022)), the effect of surface expansion by micronization might have been canceled by the decreased MAF and the increased RAF. Post-consumer PET bottles were shredded and degraded by 96.2% (approximately 40 mM) in 96 h using the best performance mutant from *T. cellulosilytica* cutinase 1 (G63A/F210I/D205C/E254C/Q93G corresponding to A109/F255/D250/E296/Q138 in Cut190). They shredded PET pieces (cut to 1 cm × 1 cm) using a high-speed rotary mill equipped with a 1.0 mm trapezoital hole sieve ring, which must have avoided repeated crush of particles with blades of the mill and suppressed the aging at minimum. Processing of PET waste into readily degradable forms, especially without sorting PET materials by shapes and crystallinity, must be considered to facilitate the practical use of PET bio-recycling. Here we would like to emphasize the importance of the micronization process of PET products to prevent aging, leading to the increase of RAF, contrary to the decrease of MAF.

We have suggested the absence of the PET binding domain in the Cut190 structure (Numoto et al. 2018), which is common to all cutinases. Furukawa et al. (2018, 2019) reported that a negatively charged surfactant promoted PET hydrolysis by *Is*PETase, and a positively charged surfactant was effective for Tf_Cut2, by increasing the binding of these enzymes on the PET surface. Rhamnolipids enhanced the hydrolysis rate, presumably by acting as mediators between *Is*PETase and PET (Gercke et al. 2021). Here, we confirmed that a cationic surfactant promoted PET hydrolysis, which can be explained by the presence of mass domains of negative amino acids on the Cut190 surface (Fig. 3B). The preference for alkyl chain length is most probably related to the appropriate distance mediated by a surfactant for the enzyme to bind on the PET surface, as illustrated in Fig. 3B. Bååth et al. (2022) reported that stronger surface binding of LCC and BTA hydrolase 1 of *T. fusca* is not optimal for the enzyme performance on PET and both enzymes showed the maximal turnover at intermediate binding strength with cetyltrimethylammonium bromide, which was in accordance with the Sabatier principle. Therefore, the optimal concentration of a surfactant must be carefully determined with concentrations of PET and enzymes. In conclusion, using an appropriate surfactant as a spacer between a solid polymer and an enzyme is simpler, less expensive, and more convenient than the fusion of outer substrate-binding domains to enable enzyme binding. In the presence of dodecyltrimethylammonium chloride, the reaction rate of Cut190*SS increased till 65 °C but decreased considerably at 67 °C, although Cut190*SS displays high activity at 70 °C (Oda et al. 2018). The activity of Cut190* against PET-S decreased at 65 °C, compared to that at 60 °C, which suggested the influence of additives included in PET-S (Oda et al. 2018). Similarly, dodecyltrimethylammonium chloride must have affected the physical property of PET (thermal aging), lowering the optimum temperature for the reaction. However, a lower reaction temperature is advantageous for saving energy. Contrary to the effect of dodecyltrimethylammonium chloride on PET hydrolysis, the surfactant showed the negative effect on PEF hydrolysis. This must be due to the difference in surface properties of PET and PEF (due to terephthalate and furanoate included in PET and PEF, respectively). Whether charge or the carbon chain length of surfactants is related to the degradation of PEF or not is remained to be further studied.

The mutation trials for the preparation of Cut190* have already been repeated, as shown in Table 1. As Cut190** showed better performance than Cut190*, Cut190**SS showed better performance than Cut190*SS. In this study, Cut190**SS/L136F possessing A138 or Cut190**SS/L136F/A138G showed the best PET conversion of approximately 90% with up to 90 mM products, although A138 seems to be better than G138 in the Cut190 structure. The position 138 (according to Cut190 sequence) is generally occurred by glutamine in homologous PET hydrolases, except LCC, which has tyrosine at this position. We noticed the importance of Q138, based on the structural analysis of Cut190 (Miyakawa et al. 2014) and first introduced a mutation at this position, replacing a large amino acid (glutamine) with a small amino acid (alanine), which resulted in better performance (Oda et al. 2018). Cut190**SS/L136F/A138G generated a slightly lower total product concentration than Cut190**SS/L136F (Cut190**SS possesses Q138A), as shown in Table 4. Tournier et al. (2020) replaced the tyrosine of LCC with glycine, not alanine, and no comparison of glycine and alanine was made. Q92 of *T. fusca* cutinase was replaced with serine, alanine and glycine, the best performance being Q92G (Chen et al. 2022). The Q138 of Cut190 is positioned in the direction of a polymer chain extension from the active site cleft (Kawabata et al. 2017), and the replacement of Q138 with a small amino acid, such as alanine or glycine, may improve the extension of the polymer chain. Further mutation to Cut190**SS/L136F/A138G/F255I as performed by Tournier et al. (2020) significantly reduced the expression, as previously found in Cut190*F255A (Oda et al. 2018). Therefore, phenylalanine at position 255 is most probably required for the stabilization of the Cut190 protein, which is completely different from the results of LCC and *T. fusca* cutinase, in which phenylalanine was mutated to isoleucine, displaying the elevated PET hydrolysis (Tournier et al. 2020; Chen et al. 2022). In addition, Cut190 has F106 (oxyanion hole-forming amino acid) and the replacement of F with Y (common to other hydrolases like TfCut2, LCC and *Is*PETase) showed less activity (Kawabata et al. 2017). Although Cut190 is homologous, especially to type II PET hydrolases, Cut190 is considered a unique PET hydrolase. The mutation of Cut190* to Cut190**SS/L136F increased the product levels (approximately 60 mM to more than 90 mM), suggesting that Cut190 shows no product inhibition. The LCC variant also accumulated an extremely high concentration of hydrolytic products in the absence of any additional enzymes able to remove MHET/BHET (Tournier et al. 2020). In the cutinase TfCut2 (Wei et al. 2016), G62A mutation was shown to release the product inhibition in PET hydrolysis. LCC and Cut190 already have alanine at this position, thereby showing no product inhibition. BHET behaved as an inhibitor for PET hydrolysis by Cut1 from *T. cellulosilytica* (Gamerith et al. 2017). As only a trace amount of BHET was detected during the reaction time (Herrero Acero et al., 2011; Barth et al. 2016; Oda et al. 2018) probably due to the fast hydrolysis of BHET (Hantani et al. 2018), significant product inhibition may not be observed with Cut190 and LCC.

Taken together, minor differences in the amino acid sequences of each cutinase are important and indispensable for each protein, although the overall structural similarity is common to Cut190, TfCut, and LCC (Kawai 2021; Kawai et al. 2019, 2020). In this study, Cut190**SS variants produced over 90 mM products (MHET and TPA) with a conversion rate of approximately 90% at 63 or 65 °C in 3 days (Table 4). Additionally, Cut190**SS produced approximately 80 mM products at 65 °C in 1 day when glycerol 24% was replaced with ethylene glycol 10% (Fig. 5). Tournier et al. (2020) amorphized and powdered post-consumer PET products on a practical scale and reached the best value of PET hydrolysis with a minimum of 90% conversion rate from 20% weight concentration of PET suspension at 72 °C in less than 1 day, using LCC variants. In our case, we used amorphous PET samples and powdered/homogenized post-consumer products on a laboratory scale. Therefore, further studies are needed, especially at a large/practical scale, to evaluate the potential application of engineered Cut190 for PET degradation. Here we used HEPES buffer, but it should be replaced with another less expensive buffer for bio-recycling as HEPES buffer is expensive for practical use in bio-recycling. Polyols are known to stabilize proteins (Vagenende et al. 2009; Naidu et al. 2020). Cut190 needs 24% glycerol to keep its activity at elevated temperature (Oda et al. 2018). Ethylene glycol showed better performance at lower concentration (6–16%) than 24% glycerol. As ethylene glycol is one of reaction products from PET or its depolymerized materials such as BHET and MHET, it might be advantageous for bio-recycling. In addition, ethylene glycol is not tenacious and easy to use, but glycerol is tenacious and a little troublesome to use. Thus, we still need improvements of Cut190 variants and their reaction conditions. Nevertheless, we can conclude that Cut190 variants would be feasible for practical application in PET recycling.

## Supplementary Information

**Additional file 1: Scheme S1. **Chemical synthesis of PEF. **Table S1.** Properties of PET samples. **Table S2.** Comparison of total products based on measurements by HPLC and absorbance at 240 nm. **Table S3.** Effect of homogenizing on crystallinity, amorphous fractions, and molecular weights. **Figure S1.** Homogenized PET samples. **Figure S2.** Powders made from amorphous PET pellets. **Figure S3.** Effect of pH and buffer. **Figure S4.** Effect of surfactants on the enzyme activity.

## Data Availability

All data generated or analyzed during this study are included in this published article and its additional information files.
