# Supplementary material for: Efficient depolymerization of polyethylene terephthalate (PET) and polyethylene furanoate by engineered PET hydrolase Cut190
Source: AMB Express. 2022 Oct 26;12:134. doi: 10.1186/s13568-022-01474-y (PMC9606173; doi:10.1186/s13568-022-01474-y)
Supplement: Supplementary file 1 — Additional file 1: Scheme S1. Chemical synthesis of PEF. Table S1. Properties of PET samples. Table S2. Comparison of total products based on measurements by HPLC and absorbance at 240 nm. Table S3. Effect of homogenizing on crystallinity, amorphous fractions, and molecular weights. Figure S1. Homogenized PET samples. Figure S2. Powders made from amorphous PET pellets. Figure S3. Effect of pH and buffer. Figure S4. Effect of surfactants on the enzyme activity. [file 13568_2022_1474_MOESM1_ESM.docx]

Supplementary information

**Efficient depolymerization of polyethylene terephthalate (PET) and polyethylene furanoate by engineered PET hydrolase Cut190**

Fusako Kawai,^1*^ Yoshitomo Furushima,^2^ Norihiro Mochizuki,^2^ Naoki Muraki,^2^ Mitsuaki Yamashita,^3^ Akira Iida,^3^ Rie Mamoto,^4^ Takehiko Tosha,^5^ Ryo Iizuka,^6^ and Sakihito Kitajima^7^

^1^ Graduate School of Environment and Life Science, Okayama University, 1-1-1 Tsushima-naka, Kita-ku, Okayama 700-8530, Japan; fkawai@okayama-u.ac.jp

^2^ Toray Research Center, Inc., 3-7 Sonoyama 3-chome, Otsu, Shiga 520-8567, Japan

^3^ Faculty of Agriculture, Kindai University, 3327-204, Nakamachi, Nara, Nara 631-8505, Japan

^4^ Division of Clinical Nutrition, Faculty of Nutrition, Kobe Gakuin University, 518 Arise, Ikawadani-cho, Nishi-ku, Kobe, Hyogo 651-2180, Japan

^5^ RIKEN SPring-8 Center, 1-1-1, Kouto, Sayo, Hyogo 679-5148, Japan

^6^ Graduate School of Sciences, University of Tokyo, 7-3-1 Hongo, Bunkyo-ku, Tokyo 113-0033, Japan

^7^ Graduate School of Science and Technology, Kyoto Institute of Technology, 1 Hashigami-cho, Matsugasaki, Sakyo-ku, Kyoto, Kyoto 606-8585, Japan

*Corresponding author:

Fusako Kawai [fkawai@okayama-u.ac.jp](mailto:fkawai@okayama-u.ac.jp)

Tel and Fax: +81-75-703-8576

***Scheme S1*.** Chemical synthesis of PEF

### The synthesis of PEF was performed using a two-stage melt-polycondensation method, as previously described by Pellis et al. (2016) with some modifications.

### A two-necked round bottom flask equipped with a distillation apparatus was used as the reaction vessel. The reaction was performed under argon atmosphere and water-free conditions. To a solution of dimethylfuran-2,5- dicarboxylate (DMDC, 1.0 g, 5.43 mmol) and 1,2-ethylene glycol (EG, 911 µL, 16.3 mmol) were added few drops of tetrabutyl titanate. The mixture was heated to 160 °C in an oil bath and stirred with a magnetic stirring bar. The temperature was kept for 2 h. Afterwards, the temperature was increased to 190 °C, and the mixture was stirred for additional 2 h. During this reaction phase, the methanol generated in the transesterification reaction of DMDC and EG was collected in the receiving flask. In the second step of polycondensation, the receiving flask was emptied to avoid foaming, whereupon the pressure in the apparatus was reduced to 0.05–0.1 mbar in a time span of 30 min. In the meantime, the temperature was increased to 220 °C and kept for 1 h. To obtain PEF with high molar mass, the temperature was increased to 235 °C, and the mixture was stirred for additional 4 h. This protocol was meant to remove the excess EG, which was collected in the receiving flask. After the apparatus was cooled down to room temperature, the resulting solid was dissolved in concentrated trifluoroacetic acid and poured into ice water. Then, the resulting precipitate was collected by suction filtration, and the cake was washed with MeOH prior to drying. The lower Mw PEF (approximately 21 kDa) was obtained as white powders and the higher Mw PEF (approximately 74 kDa) as brownish brittle powder. The chemical structure of PEF was confirmed by ^1^H NMR and 13C NMR. ^1^H NMR (400MHz, CF_3_CO_2_D): δ = 7.41 (2H, t, *J* = 4.9 Hz), 3.24 (2H, t, *J* = 4.9 Hz). ^13^C NMR (100MHz, CF_3_CO_2_D): δ = 160.7, 146.6, 120.5, 64.2. ^1^H NMR and ^13^C NMR spectra were recorded on a Bruker AVANCE-III 400 spectrophotometer.

***Table S1.*** Properties of PET samples

| PET sample | *T*g (°C) | Crystallinity (%) | MAF (%) (A) | RAF (%) (B) | (A)+(B) |
| --- | --- | --- | --- | --- | --- |
| PET film: | | | | | |
| PET-GF | 73 | 3* | 89 | 8* | 97 |
| NOACRYSTAL-V | 74 | 0* | 91 | 9* | 100 |
| NOACRYSTAL-R | 74 | 0* | 89 | 11* | 100 |
| Package: before homogenizing | | | | | |
| Food package 1 | 74 | 0* | 91 | 9* | 100 |
| Food package 2 | 74 | 1* | 89 | 10* | 99 |
| PET bottle | 88 | 21 | 34 | 45 | 79 |
| Waste flakes  (from PET bottles) | 107 | 28 | 33 | 39 | 72 |
| PET-S | 76 | 4* | 92 | 4* | 96 |
| Powder: | | | | | |
| Powder 1 | 78 | 2* | 86 | 12* | 98 |
| Powder 2 | 78 | 5* | 87 | 8* | 95 |
| Powder 3 | 82 | 0* | 79 | 21* | 100 |

*T*_g_: glass transition temperature. MAF: mobile amorphous fraction. RAF: rigid amorphous fraction. Powders 1 and 3 were milled, using the same PET amorphous pellet.

Measurements of *T*_g_, crystallinity, MAF, and RAF were performed by differential scanning calorimetry (DSC) using a Q100 from TA Instruments Japan Inc. (Tokyo). Dry nitrogen gas with a flow rate of 50 ml min^-1^ was purged through the cell. The sample mass was 5 mg. The crystallinity (*X*_cryst._) was calculated by the standard DSC measurement at a heating rate of 10 ^○^C min^-1^ as follows:

*X*_cryst._ = (Δ*H*_m_ /Δ*H*_cryst._) ×100 (1)

where Δ*H*_m_ is the observed heat of fusion that is estimated by extrapolating a linear baseline from the molten state, and Δ*H*_cryst._ (= 140.1 J g^-1^) is the heat of fusion for perfect crystallinity PET (Wunderlich B 2005).

The mobile amorphous fraction (*X*_MAF_) was determined by using temperature modulated DSC (TMDSC) experiment as follows:

*X*_MAF_ = (Δ*C*_p_ /Δ*C*_p, amorph._) ×100 (2)

where Δ*C*_p_ is the observed heat capacity change at *T*_g_, and Δ*C*_p, amorp._ (= 0.4052 J g^-1^ K^-1^) is the heat capacity change at *T*_g_ for perfect amorphous PET (Wunderlich B 2005). Here, the heating rate, temperature amplitude, and modulation period of TMDSC measurements were 2 ^○^C min^-1^, 1 ˚C, and 60 s, respectively.

The rigid amorphous fraction (*X*_RAF_) was determined as follows:

*X*_RAF_ = 100- *X*_cryst._- *X*_MAF_ (3)

* Contains a few percent error due to difficulty in distinguishing between cold crystallization and melting signals on a DSC trace. Zero % crystallinity of amorphous PET was reported by Kong and Hay (2003).

***Table S2.*** Comparison of total products based on measurements by HPLC and absorbance at 240 nm

| Enzyme reaction | Total products (mM)  Based on HPLC at 240 nm | Total products (mM)  Based on absorbance at 240 nm |
| --- | --- | --- |
| 0-day | 0.0 | 0.0 |
| 1-day | 15.3±0.1 | 16.1±0.1 |
| 2-day | 27.9±0.6 | 30.8± 0.1 |
| 3-day | 38.5±1.1 | 42.3± 0.9 |

The reaction mixture contained 0.15 M HEPES buffer (pH 9.0), 24% glycerol, 2.5 mM CaCl_2_ and 2 μM Cut190*SS, 1 piece of 6mm ∅ NOACRYSTAL-V in total 500 μl. Incubation was carried out at 63 °C with shaking. An aliquot (25 μl) of reaction mixtures was withdrawn and diluted by 25 μl of methanol. If necessary, dilution was repeated. The molar extinction value (ε) of TPA in 50% methanol was 16.8×10^3^ M^-1^ cm^-1^.

***Table S3*.** Effect of homogenizing on crystallinity, amorphous fractions, and molecular weights

| PET sample | *T*_g_  (°C) | Crystallinity (%) | MAF (%) (A) | RAF (%) (B) | (A)+(B)  (%) | Mn | Mw | Mn/Mw |
| --- | --- | --- | --- | --- | --- | --- | --- | --- |
| Waste pellets from PET bottles | | | | | | | | |
| Before homogenizing | 107 | 28 | 33 | 39 | 72 | 8,290 | 23,600 | 2.85 |
| After homogenizing | 81 | 28 | 29 | 43 | 72 | 8,670 | 24,700 | 2.85 |
| Amorphous film | | | | | | | | |
| Before homogenizing | 75 | 0 | 88 | 12 | 100 | 6,260 | 18,000 | 2.88 |
| After homogenizing | 82 | 1 | 44 | 55 | 99 | 6,240 | 18,000 | 2.88 |

Mn: number-average molecular weight. Mw: weight-average molecular weight.

1. Waste PET bottle flakes B. PET bottle


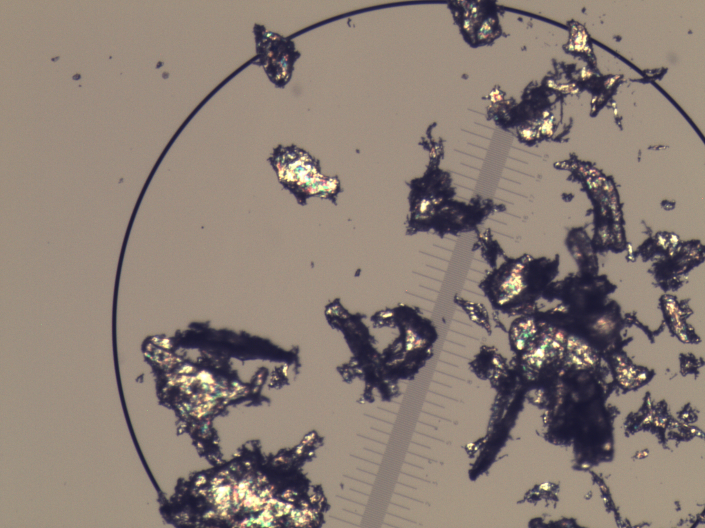

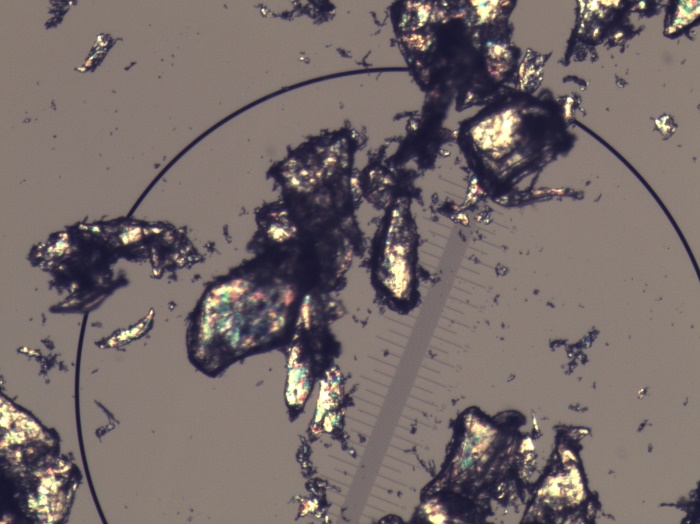


C. Amorphous PET film D. Food package


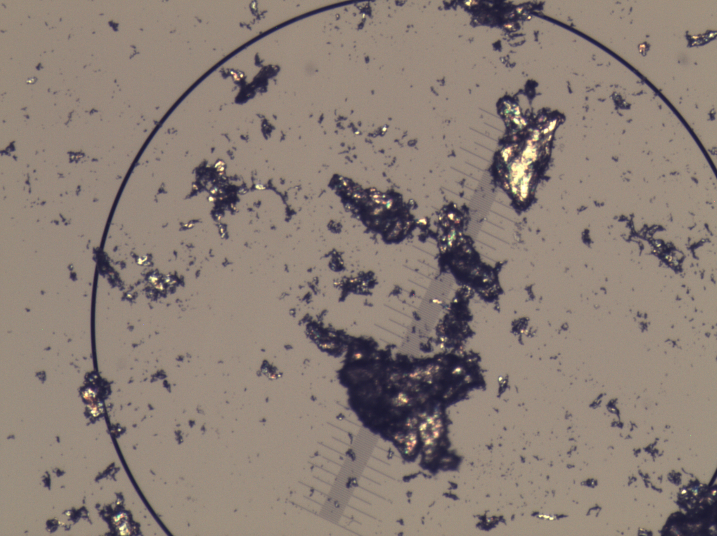

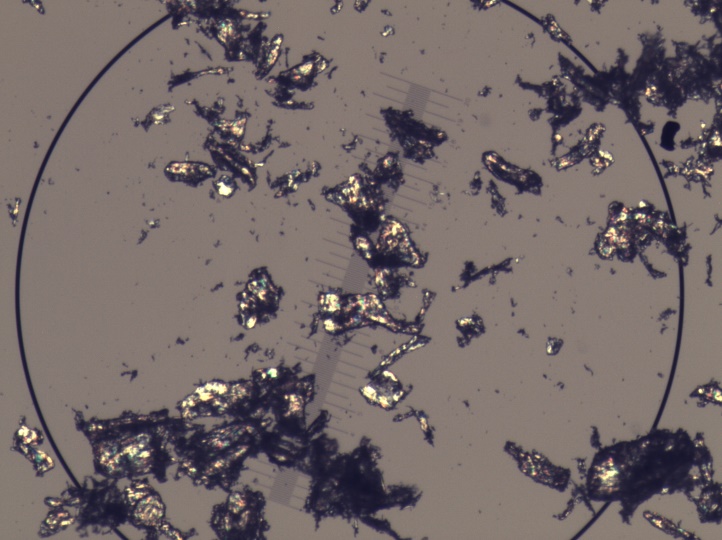

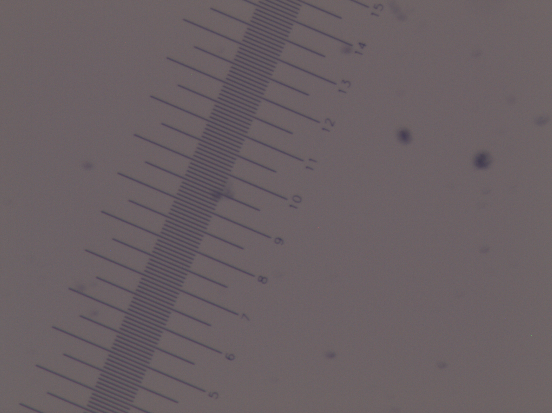


***Figure S1.*** Homogenized PET samples. One scale number corresponds to 10 μm.

1. Powder 1 B. Powder 2 C. Powder 3


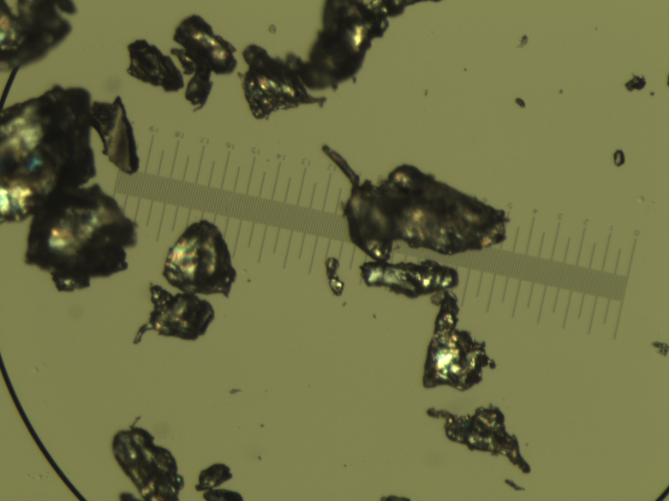

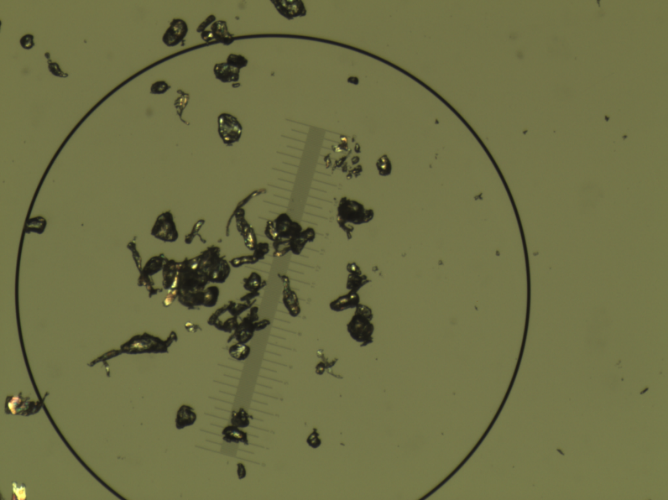

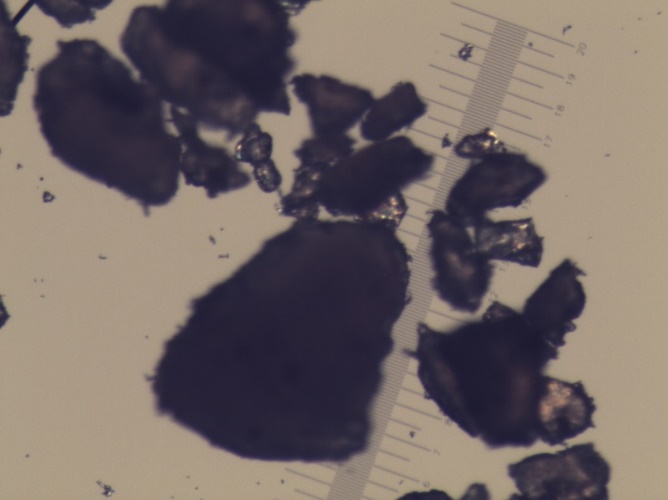


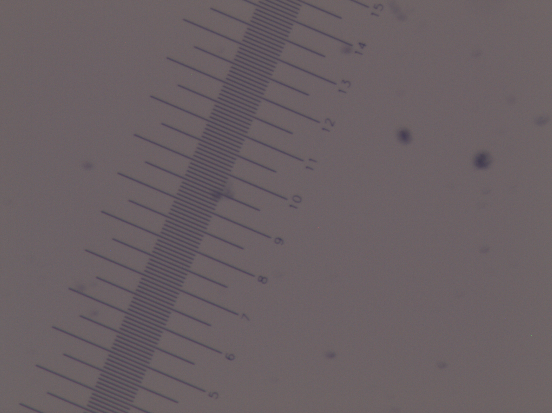


***Figure S2.***　Powders made from amorphous PET pellets. One scale number corresponds to 10 μm.

***Figure S3.*** Effect of pH and buffer

The reactions were performed at 65 °C for 7 days, using Cut190*SS, 0.1 M buffer and one piece of 6 mm ∅ PET-GF film in a 500 μl of reaction mixture, as described in Materials and Methods.

***Figure S4.*** Effect of surfactants on the enzyme activity. The reactions were performed at 65 °C for 2 days, using Cut190*SS, 100 mM HEPES buffer (pH 8.5), 30 ppm surfactant and one piece of 6mm ∅ PET-GF film in a 1-ml reaction mixture, as described in Materials and Methods. The reaction mixtures except the enzyme and CaCl_2_ were preincubated at room temperature for 15−30 min to allow the sufficient binding of surfactant on the surface of PET film. Then the enzyme and CaCl_2_ were added, and the reaction was started at 65 °C with shaking. Surfactants used were Tween 20 (polyoxyethylene sorbitan monolaurate) (nonionic), dodecylammonium chloride (cationic) and sodium dodecyl sulfate (anionic). Surfactants used have no specific absorbance at 240 nm and non-specific absorbance at 240 nm was negligible below 100 ppm.

**References**

Kong Y, Hay JN (2003) The enthalpy of fusion and degree of crystallinity of polymers as measure by DSC. Eur Polym J 39: 1721-1727. doi: 10.1016/S0014-3057(03)00054-5

Wunderlich B (2005) Thermal analysis of polymeric materials. Springer, Berlin
